# Age and hypoalbuminemia independently predict pulmonary consolidation in children with 23S rRNA A2063G-mutant *Mycoplasma pneumoniae* pneumonia: a retrospective single-center study

**DOI:** 10.3389/fped.2026.1773231

**Published:** 2026-05-21

**Authors:** Haiwen Guo, Xin Zhang, Lirong Luo

**Affiliations:** 1 Department of Pediatrics, Guangzhou Red Cross Hospital of Jinan University, Guangzhou, China; 2 Department of Cardiac Surgery Intensive Care Unit, Guangdong Provincial People’s Hospital (Guangdong Academy of Medical Sciences), Southern Medical University, Guangzhou, China; 3 Department of Pediatrics, Guangzhou Red Cross Hospital of Jinan University, Guangzhou, China

**Keywords:** 23S rRNA a2063G mutation, mycoplasma pneumoniae, peripheral blood composite indices, pulmonary consolidation, risk factors

## Abstract

**Background:**

*Mycoplasma pneumoniae* pneumonia (MPP) with 23S rRNA A2063G mutation is prone to progressive pulmonary consolidation. This study aimed to explore the clinical characteristics and independent risk factors of pulmonary consolidation among such children.

**Methods:**

A retrospective, single-center analysis was conducted on the clinical data of children with MPP admitted to a Grade A tertiary general hospital between January 2023 and December 2024. Based on targeted next-generation sequencing (tNGS), children were divided into A2063G mutation-positive and -negative groups. Mutation-positive children were further classified into pulmonary consolidation and non-consolidation subgroups. Multivariate logistic regression was used to identify independent risk factors for pulmonary consolidation in A2063G mutation-positive MPP children.

**Results:**

A total of 347 children were included in the study. Compared with children without the A2063G mutation, children with the mutation presented older age, higher rates of pre-admission cough and pulmonary consolidation, as well as elevated globulin (GLB), neutrophil-to-lymphocyte ratio (NLR), monocyte-to-lymphocyte ratio (MLR), platelet-to-lymphocyte ratio (PLR), pan-immune-inflammation value (PIV) and systemic immune-inflammation index (SII) levels; meanwhile, their lymphocytes (LYM) and albumin (ALB) levels were significantly lower (all *P* < 0.05). Within children with the A2063G mutation, those with pulmonary consolidation had higher duration of fever, maximum body temperature, length of hospital stay, severe pneumonia rate, and C-reactive protein-to-lymphocyte ratio (CLR), but lower rate of bilateral lung inflammation, ALB, and uric acid (UA) than those without pulmonary consolidation (all *P* < 0.05). Multivariate logistic regression analysis identified age and ALB as independent risk factors for pulmonary consolidation in A2063G mutation-positive MPP children (OR =  1.010 and 0.875, respectively; both *P* < 0.05).

**Conclusion:**

Older age and lower ALB levels are associated with a higher risk of pulmonary consolidation in children with A2063G-mutant MPP, suggesting that an enhanced host inflammatory immune response contributes to the development of pulmonary consolidation.

## 1 Introduction

*Mycoplasma pneumoniae* (MP) is one of the leading pathogens of community-acquired pneumonia (CAP) in children. *Mycoplasma pneumoniae* pneumonia (MPP) shows periodic epidemic patterns, with peaks occurring approximately every 3–7 years, posing a substantial threat to pediatric health (1). In recent years, multiple regions in China have reported a surge in MPP, drawing considerable attention (2).

The clinical manifestations of MPP are diverse, ranging from self-limiting upper respiratory tract infections to severe cases that progress to refractory pneumonia, complicated by consolidation and other serious pulmonary lesions. Pulmonary consolidation, a critical complication of MPP, is pathologically characterized by alveolar exudation and parenchymal involvement. It can impair ventilation, cause persistent high fever and respiratory distress, increase the need for mechanical ventilation and intensive care unit (ICU) admission, and prolong hospital stays. Moreover, some children may develop long-term sequelae such as atelectasis, bronchiectasis, or interstitial fibrosis, which are linked to an elevated risk of adverse outcomes (3). Therefore, early identification of patients at high risk of consolidation is essential for improving prognosis.

In terms of treatment, the development of pulmonary consolidation necessitates a more complex therapeutic strategy, primarily involving the appropriate selection of antiviral agents or antibiotics based on pathogen characteristics, as well as supportive care such as antipyretic therapy and oxygen therapy (3, 4). According to relevant pneumonia guidelines, glucocorticoids are recommended only for patients with severe or critical pneumonia rather than for all those with concurrent pulmonary consolidation; intravenous immunoglobulin, as an immunomodulatory therapy, is also recommended only for patients with extrapulmonary complications such as central nervous system involvement, not for children with isolated pulmonary consolidation (4). In conventional treatment, macrolide antibiotics were once the first-line agents; however, their clinical efficacy has declined significantly with the widespread spread of drug-resistant strains (4). Accumulating evidence has confirmed that macrolide resistance is mainly associated with mutations in the 23S rRNA gene, among which A2063G is the most common (4). The reported prevalence of macrolide-resistant *Mycoplasma pneumoniae* (MRMP) in Asia is approximately 69%–95%, while recent studies from China have shown rates as high as 90.0%–98.9%, often with regionally clustered patterns (5–7).

Previous studies suggest that drug-resistant MPP are associated with prolonged disease course, heightened inflammatory response, and increased radiological severity (8, 9). Nevertheless, whether the A2063G mutation represents an independent risk factor for pulmonary consolidation remains controversial. Some scholars argue that it worsens the disease mainly through indirect mechanisms, such as delayed treatment or amplified immune responses, while its direct pathogenic role has yet to be systematically established (10). Against this background, the present study aimed to characterize the clinical features of pulmonary consolidation and identify risk factors among children with MPP carrying the A2063G mutation, with the goal of establishing independent predictive indicators to guide early risk assessment and individualized treatment.

## 2 Methods

### 2.1 Study design

This was a single-center retrospective study. Using the electronic medical record system of Guangzhou Red Cross Hospital of Jinan University, we collected clinical data of children hospitalized with CAP between January 2023 and December 2024. Consecutive children admitted during the study period who met the diagnostic criteria for pediatric CAP (11) were enrolled as study participants. At our institution, targeted next-generation sequencing (tNGS) is routinely performed as an etiological examination for pediatric CAP, and tNGS testing was conducted in all enrolled children. Accordingly, only children with a tNGS-confirmed diagnosis of MPP were ultimately included in the analysis. To minimize selection bias and information bias, which are inherent in retrospective studies, case screening was performed via double-independent verification. Patient data were separately verified by two researchers, and any discrepancies were adjudicated by a third senior researcher.

### 2.2 Study participants

The inclusion criteria were as follows: ① age ≥ 28 days and < 18 years; ② meeting the diagnostic criteria for pediatric CAP; ③ positive for MP infection as detected by throat swab tNGS. The exclusion criteria were: ① comorbidities with underlying diseases such as congenital heart disease, immunodeficiency, or hematological malignancies; ② presence of chronic lung diseases (e.g., bronchopulmonary dysplasia, bronchial asthma); ③ lack of informed consent (as required by the institutional review board) or incomplete clinical data; ④ hospitalization duration <24 h or voluntary discharge against medical advice.

### 2.3 tNGS sample testing methods

#### 2.3.1 tNGS sample collection and transport

A throat swab or lower respiratory tract specimen (e.g., tracheal aspirate, bronchoalveolar lavage fluid) was collected from all children on the day of admission. Following oral rinsing 2–3 times with normal saline prior to sampling, throat swabs were collected and immediately placed into MT0301-6 viral transport tubes for sealing and transportation on dry ice at −20 °C to KingMed Diagnostic Center in Guangzhou, China for testing (12). Lower respiratory specimens were obtained via bronchoscopy as BALF. The primary indications for this procedure included severe or refractory pneumonia with unknown etiology and poor response to conventional treatment, as well as pulmonary lesions requiring further etiological clarification (13).

#### 2.3.2 tNGS testing platform and process

Target fragments were amplified using the KS608-100HXD96 Respiratory Pathogen Detection Kit of Guangzhou Jinqirui Biotechnology via multiplex polymerase chain reaction (PCR), followed by library construction. And tNGS was performed on the KMMiniSeqDx-CN sequencing platform. The assay covered common respiratory pathogens and mutations in domain *V* of the 23S rRNA gene associated with macrolide resistance in MP, including A2063G, A2064G, A2067G, and C2617G, with a lowest limit of detection of 100 copies/mL.

#### 2.3.3 Mutation interpretation and quality control

Sequencing data was analyzed by a third-party medical testing laboratory using standardized bioinformatics workflows. Identification of drug-resistant mutations was based on the proportion of mutant sequences relative to total sequences at the target loci (i.e., mutation frequency). When both wild-type and mutant sequences coexisted in a sample, a comprehensive interpretation was performed combined with mutation frequency. Internal controls, positive controls, and negative controls were included throughout the experiment, and sequencing quality indicators (e.g., the proportion of Q30 bases) met the laboratory's quality control standards to ensure the reliability of the test results.

#### 2.3.4 Coinfection determination and differentiation of colonization vs. infection

tNGS enables simultaneous detection of multiple respiratory pathogens. For samples with multiple pathogens identified, a comprehensive analysis was conducted by integrating normalized sequence counts, estimated microbial concentrations (copies/mL), and pathogenicity classification (Classes A/B/C). These findings were further combined with the children' s clinical manifestations, imaging features, and other laboratory results to distinguish between infection and colonization. When necessary, results were validated using alternative assays such as culture and PCR or by testing specimens from different anatomical sites.

### 2.4 Evaluation of imaging data

All imaging examinations and diagnoses for the children were completed prior to the availability of tNGS results. The data were independently reviewed by a senior radiologist and a pediatrician, both blinded to the their genetic mutation status and clinical severity; any disagreements were resolved by a third expert to ensure the objectivity and accuracy of the assessment. CXR findings were categorized as bronchopneumonia and consolidation/atelectasis, whereas CCT findings were categorized as bronchopneumonia, consolidation/atelectasis, bronchitis, and mosaic pattern (14). Based on this classification, the definitions of pulmonary consolidation and atelectasis were individually further refined. Pulmonary consolidation was defined radiologically as homogeneous increased density of the lung parenchyma obscuring vascular and airway borders, with or without air bronchograms, usually involving one or more pulmonary segments or lobes, without evidence of decreased lung volume (15). Atelectasis was defined as increased lung tissue density accompanied by reduced lung volume, manifested as interlobar fissure displacement, mediastinal shift, or retraction of adjacent structures (16). The distinction between pulmonary consolidation and atelectasis was based mainly on the presence or absence of decreased lung volume. For cases that cannot be clearly classified, the final classification is determined through discussion and voting among experienced radiology and pediatrics specialists to minimize misclassification bias and ensure the objectivity as well as accuracy of the results.

### 2.5 Criteria for diagnosing severe pediatric pneumonia

Diagnostic criteria were based on the Chinese Guidelines for the Management of Community-Acquired Pneumonia in Children (2024 Revision) (11), and severe pneumonia was defined as the presence of any one of the following manifestations: ① poor general condition;② refusal to eat or signs of dehydration; ③ altered mental status; ④ markedly increased respiratory rate (infants ≥70 breaths/min, older children ≥50 breaths/min); ⑤ dyspnea; ⑥ cyanosis; ⑦ oxygen saturation ≤0.92 (at sea level) or <0.90 (at high altitude); ⑧ pulmonary infiltrates involving multiple lobes or ≥2/3 of a single lobe; ⑨ pleural effusion; ⑩ extrapulmonary complications.

### 2.6 Data collection

Clinical, imaging, and laboratory data were collected from all enrolled children. All assessments and examinations were completed on the day of admission and based on initial test results.

Clinical characteristics included sex, age, duration of fever, peak temperature, fever before admission, cough before admission, time from disease onset to macrolide use, rales and location of it, co-infection, severe pneumonia, and length of hospital stay. Imaging data comprised CXR and/or CCT, focusing on the location of pneumonia and the presence of pulmonary consolidation.

Laboratory parameters were classified into three categories: pathogen detection marker, inflammatory and immune-related markers, and other laboratory indicators, with the latter two belonging to hematological markers. ① Pathogen detection marker is tNGS using throat swab specimen. ② Inflammatory and immune-related markers: white blood cells (WBC), neutrophils (NEU), lymphocytes (LYM), monocytes (MONO), eosinophils (EOS), C-reactive protein (CRP), procalcitonin (PCT), and interleukin-6 (IL-6). Based on the above indicators, several composite peripheral blood inflammatory indices were calculated, including the neutrophil-to-lymphocyte ratio (NLR), monocyte-to-lymphocyte ratio (MLR), platelet-to-lymphocyte ratio (PLR), C-reactive protein-to-lymphocyte ratio (CLR), pan-immune-inflammation value (PIV) = (NEU × MONO × PLT)/LYM, and systemic immune-inflammation index (SII) = (NEU × PLT)/LYM. ③ Other laboratory markers: hematological markers: red blood cells (RBC), hemoglobin (HB), platelets (PLT); cardiac markers: creatine kinase (CK), creatine kinase-MB (CKMB), lactate dehydrogenase (LDH); liver function markers: alanine aminotransferase (ALT), aspartate aminotransferase (AST), total protein (TP), albumin (ALB), globulin (GLB); renal function markers: blood urea nitrogen (BUN), creatinine (Cr), uric acid (UA).

The primary outcome measures investigated were clinical characteristics, imaging findings, and inflammatory and immune-related markers. To ensure the accuracy and reliability of data entry, all data were independently recorded by two researchers and cross-checked. Any inconsistencies were adjudicated by a third researcher to determine the final results.

### 2.7 Data analysis

Statistical analyses were performed using SPSS 28.0 software (International Business Machines Corporation, Armonk, New York, United States). The normality of continuous variables was assessed using the Shapiro–Wilk test. Normally distributed data were presented as mean ± standard deviation (*x¯* ± *s*) and compared between groups using the independent-samples *t*-test, with Cohen's *d* reported as the effect size. Non-normally distributed data were expressed as median (interquartile range, M [P25, P75]) and compared using the Mann–Whitney U test, with the r value reported as the effect size. Categorical variables were presented as numbers (percentages) and compared using the *χ*^2^ test or Fisher's exact test; the Phi coefficient or Cramer's *V* was calculated to evaluate the effect size.

Multivariate logistic regression analysis was performed to identify independent factors associated with pulmonary consolidation. Variables with *P* < 0.10 in univariate analysis were entered into the regression model, and odds ratios (OR) with corresponding 95% confidence intervals (95% CI) were calculated. Before modeling, multicollinearity among independent variables was evaluated using the variance inflation factor (VIF) and tolerance. Receiver operating characteristic (ROC) curves were constructed based on predicted probabilities from the logistic regression model. The area under the curve (AUC), sensitivity, and specificity were calculated to assess the discriminatory performance of the model, and the optimal cutoff value was determined according to the Youden index. A *P*-value <0.05 was considered statistically significant.

### 2.8 Ethics statement

This study was conducted in strict accordance with the principles of the Declaration of Helsinki and was approved by the Ethics Review Committee of Guangzhou Red Cross Hospital of Jinan University (Approval No. 2025-331-01). As this was a retrospective study, the Ethics Committee granted a waiver of written informed consent. Privacy protection regulations were strictly followed during the study, and all collected data were anonymized to safeguard the personal information and rights of the participants.

## 3 Results

### 3.1 Patient characteristics

**Figure 1:**
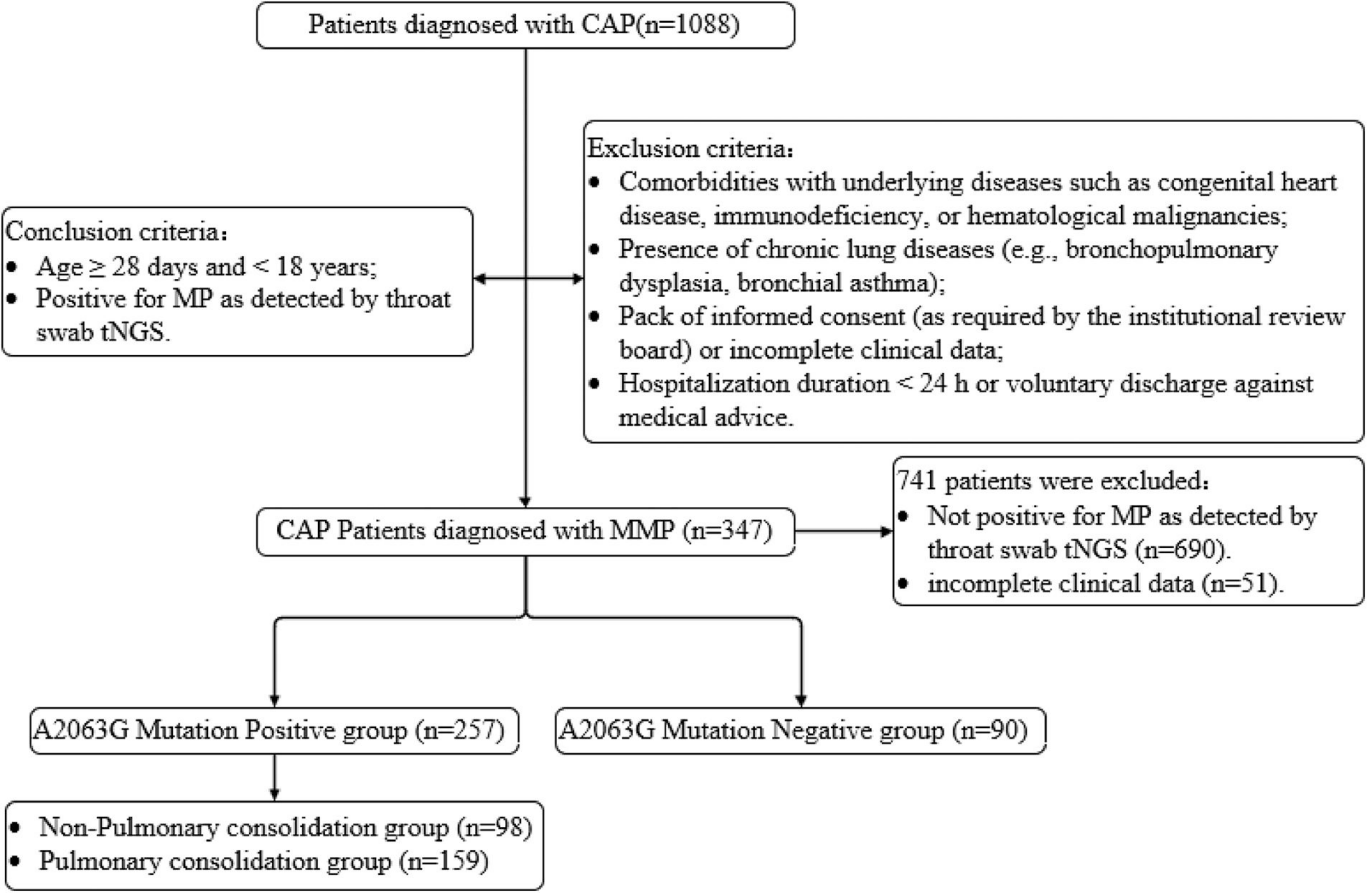
Participant selection process for pediatric patients with MPP.

A total of 1,088 children were admitted to the general pediatric ward and diagnosed with CAP. After screening, 741 cases were excluded (including 690 cases with negative MP results on tNGS testing and 51 cases with incomplete clinical data). Ultimately, 347 eligible children were included in the study. A detailed flowchart of the participant selection process is shown in Figure 1. Of these 347 children, 182 were male (52.5%) and 165 were female (47.6%), with a male-to-female ratio of 1.10:1. All children underwent tNGS testing, including 336 throat swabs (96.8%) and 11 bronchoalveolar lavage fluid (BALF) samples (3.2%). Radiological examinations were performed as follows: 278 children (80.1%) underwent chest x-ray (CXR) only, 6 children (1.7%) underwent chest computed tomography (CCT) only, and 63 children (18.2%) received both CXR and CCT examinations.

### 3.2 Univariate analysis of clinical and imaging features between the A2063G mutation-negative group and mutation-positive group in MPP children

According to throat swab tNGS results, the macrolide-resistant locus in MPP was exclusively identified as A2063G. Among the 347 enrolled children, 90 (26%) were A2063G mutation-negative and 257 (74.1%) were mutation-positive. Compared with the mutation-negative children, the mutation-positive children had significantly higher median age, pre-admission cough rate, and pulmonary consolidation rate (all *P* < 0.05). No significant differences were found in these children in the two groups regarding gender, duration of fever, peak temperature, pre-admission fever, time from onset to macrolide administration, pulmonary rales, pneumonia location, co-infection, severe pneumonia, or length of hospital stay (all *P* > 0.05) (Table 1).

**Table 1:** Univariate analysis of clinical and imaging features between the A2063G mutation-negative group and mutation-positive group in MPP children (*n* = 347).

Variables	Negative group (*n* = 90)	Positive group (*n* = 257)	*Z*/*χ*^2^ value	*P* value	Effect size
Sex, *n* (%)			0.003	0.960	−0.003
Male	47 (52.2)	135 (52.5)			
Female	43 (47.8)	122 (47.5)			
Age(months)	74.50 (50.75, 94.75)	85.00 (60.00, 109.00)	−2.544	**<0.05**	−0.137
Duration of fever (days)	6.00 (4.00, 7.00)	6.00 (4.00, 7.00)	−0.846	0.398	−0.045
Peak temperature ( °C)	39.20 (38.50, 39.80)	39.30 (38.80, 39.80)	−0.432	0.665	−0.023
Fever before admission, *n* (%)			0.116	0.734	−0.018
No	7 (7.8)	23 (8.9)			
Yes	83 (92.2)	234 (91.1)			
Cough before admission, *n* (%)				**<0.05**	0.158
No	3 (3.3)	0 (1.7)			
Yes	87 (96.7)	257 (100)			
Time from onset to macrolide use (days)	6.00 (4.00, 7.00)	5.00 (3.00, 7.00)	−0.954	0.340	−0.051
Rales, *n* (%)			0.815	0.367	0.048
No	6 (6.7)	11 (4.3)			
Yes	84 (93.3)	246 (95.7)			
Site of pneumonia, *n* (%)			0.696	0.706	0.045
Left	21 (23.3)	66 (25.7)			
Right	37 (41.1)	93 (36.2)			
Bilateral	32 (35.6)	98 (38.1)			
Pulmonary consolidation, *n* (%)			5.520	<**0**.**05**	0.126
No	68 (75.6)	159 (61.9)			
Yes	22 (24.4)	98 (38.1)			
Co-infection, *n* (%)			2.510	0.474	0.085
None	35 (38.9)	115 (44.7)			
Bacterial	25 (27.8)	71 (27.6)			
Viral	13 (14.4)	39 (15.2)			
Multiple	17 (18.9)	32 (12.5)			
Severe pneumonia, *n* (%)			0.549	0.459	−0.400
No	56 (62.2)	171 (66.5)			
Yes	34 (37.8)	86 (33.5)			
Length of hospital stay (days)	9.00 (7.00, 12.50)	9.00(8.00, 11.00)	−0.362	0.718	−0.019

Note: Fisher's exact test was performed as the assumption of *χ*^2^ test was violated (one expected count <1).

Bold values indicate statistically significant variables (*P* <0.05).

### 3.3 Univariate analysis of hematological indicators between the A2063G mutation-negative group and mutation-positive group in MPP children

The mutation-positive children had significantly lower LYM and ALB than the mutation-negative children (all *P* < 0.05). Significantly higher globulin GLB, NLR, MLR, PLR, PIV, and SII were observed in the mutation-positive children compared with the mutation-negative children (all *P* < 0.05). Although the differences in these indicators among the children were statistically significant, the magnitude of these differences was relatively limited. No significant differences were found in the children in the two groups regarding WBC, RBC, HB, PLT, NEU, MONO, EOS, CRP, PCT, IL6, LDH, CK, CKMB, ALT, AST, TP, BUN, Cr, UA, or CLR (all *P* > 0.05) (Table 2).

**Table 2:** Univariate analysis of hematological indicators between the A2063G mutation-negative group and mutation-positive group in MPP children (*n* = 347).

Variables	Negative group (*n* = 90)	Positive group (*n* = 257)	*t/Z value*	*P value*	Effect size
WBC (×10^9^/L)	7.62 (6.15, 8.73)	7.28 (5.87, 8.89)	−0.501	0.617	−0.027
NEU (×10^9^/L)	4.34 (3.19, 5.33)	4.61 (3.42, 5.66)	−1.394	0.163	−0.075
LYM (×10^9^/L)	2.18 (1.65, 2.94)	1.91 (1.39, 2.51)	−2.792	**<0** **.** **05**	−0.150
MONO (×10^9^/L)	0.58 (0.42, 0.76)	0.56 (0.41, 0.78)	−0.049	0.961	−0.003
EOS (×10^9^/L)	0.06 (0.02, 0.18)	0.06 (0.03, 0.15)	−0.248	0.804	−0.013
CRP (mg/L)	11.65 (6.07, 24.90)	10.90 (5.70, 23.15)	−0.026	0.980	−0.001
PCT (ng/mL)	0.12 (0.08, 0.20)	0.11 (0.06, 0.18)	−1.382	0.167	−0.074
IL6 (pg/mL)	16.57 (11.53, 23.36)	17.04 (10.27, 24.57)	−0.013	0.989	−0.001
NLR	1.76 (1.22, 2.89)	2.38 (1.66, 3.32)	−3.22	**<0** **.** **05**	−0.173
MLR	0.27 (0.18, 0.33)	0.29 (0.21, 0.38)	−2.211	**<0** **.** **05**	−0.119
PLR	116.20 (85.35, 153.08)	143.52 (102.74, 185.86)	−3.197	**<0** **.** **05**	−0.172
CLR	5.06 (2.37, 12.89)	6.06 (2.65, 14.16)	−1.195	0.232	−0.064
PIV	278.44 (158.22, 460.80)	328.28 (195.06, 575.15)	−2.191	**<0** **.** **05**	−0.118
SII	467.35 (328.04, 726.51)	616.16 (403.33, 947.93)	−2.912	**<0** **.** **05**	−0.156
RBC (×10^12^/L)	4.54 (4.35, 4.77)	4.57 (4.35, 4.88)	−0.667	0.505	−0.036
HB (g/L)	123.50 (117.00, 130.00)	125.00 (119.00, 131.00)	−1.090	0.276	−0.059
PLT (×10^9^/L)	264.50 (206.75, 338.50)	265.00 (214.00, 323.50)	−0.117	0.907	−0.006
LDH (U/L)	324.20 (274.35, 363.40)	304.90 (269.65, 353.55)	−1.418	0.156	−0.076
CK (U/L)	107.40 (77.67, 145.32)	111.30 (78.75, 164.50)	−0.579	0.563	−0.031
CKMB (U/L)	22.95 (18.95, 29.80)	22.80 (18.80, 28.55)	−0.036	0.971	−0.002
ALT (U/L)	13.20 (10.60, 17.23)	13.80 (11.10, 17.10)	−0.565	0.572	−0.030
AST (U/L)	30.60 (27.18, 36.17)	30.60 (25.70, 36.60)	−0.792	0.428	−0.043
TP (g/L)	69.50 (66.83, 72.55)	68.80 (67.00, 71.40)	−0.826	0.409	−0.044
ALB (g/L)	42.44 ± 3.21	41.03 ± 2.74	4.019	**<0** **.** **05**	0.472
GLB (g/L)	26.40 (24.38, 28.88)	27.90 (25.80, 30.65)	−2.733	**<0** **.** **05**	−0.147
BUN (mmol/L)	3.10 (2.60, 3.60)	3.30 (2.60, 3.90)	−1.292	0.196	−0.069
Cr (*μ*mol/L)	36.97 ± 9.09	36.96 ± 9.35	0.003	0.998	0.001
UA (μmol/L)	248.00 (201.00, 290.25)	263.80 (206.40, 314.50)	−1.716	0.086	−0.092

Bold values indicate statistically significant variables (*P* <0.05).

### 3.4 Univariate analysis of clinical and imaging features between the non-consolidation group and consolidation group in A2063G mutation-positive MPP children

Of the 257 A2063G mutation-positive children, 159 (61.9%) were classified into the non-consolidation group and 98 (38.1%) into the consolidation group. Compared with the non-consolidation children, the consolidation children showed significantly higher age, duration of fever, peak temperature, length of hospital stay, and rate of severe pneumonia (all *P* < 0.05). There was also a statistically significant difference in the site of pneumonia among the children (*P* < 0.05). Furthermore, no significant differences were found in these children regarding gender, fever before admission, time from onset to macrolide administration, rales, or co-infection (all *P* > 0.05) (Table 3).

**Table 3:** Univariate analysis of clinical and imaging features between the non-consolidation group and consolidation group in A2063G mutation-positive MPP children (*n* = 257).

Variables	Non-consolidation group (*n* = 159)	Consolidation group (*n* = 98)	*Z/χ*^2^ *value*	*P value*	Effect size
Sex, *n* (%)			0.406	0.524	0.040
Male	86 (54.1)	49 (50.0)			
Female	73 (45.9)	49 (50.0)			
Age (months)	80.00 (56.00, 104.00)	91.00 (72.50, 115.00)	−2.879	**<0** **.** **05**	−0.155
Duration of fever (days)	6.00 (4.00, 7.00)	7.00 (5.00, 8.00)	−2.897	**<0** **.** **05**	−0.156
Peak temperature ( °C)	39.10 (38.50, 39.60)	39.50 (39.00, 39.92)	−2.282	**<0** **.** **05**	−0.123
Fever before admission, *n* (%)			1.554	0.213	0.078
No	17 (10.7)	6 (6.1)			
Yes	142 (89.3)	92 (92.9)			
Time from onset to macrolide use (days)	5.00 (3.00, 7.00)	5.00 (3.00, 7.00)	−1.330	0.183	−0.071
Rales, *n* (%)			0.261	0.609	−0.032
No	6 (3.8)	5 (5.1)			
Yes	153 (96.2)	93 (94.9)			
Site of pneumonia, *n* (%)			31.355	<0.001	0.349
Left	37 (23.3)^a^	29 (29.6)^a^			
Right	41 (25.8)^a^	52 (53.1)^a^			
Bilateral	81 (50.9)^b^	17 (17.3)^b^			
Co-infection, *n* (%)			4.417	0.220	0.131
None	77 (48.4)	38 (38.8)			
Bacterial	39 (24.5)	32 (32.7)			
Viral	21 (13.2)	18 (18.4)			
Multiple	22 (13.8)	10 (10.2)			
Severe pneumonia, *n* (%)			50.873	<**0**.**05**	0.445
No	132 (83.0)	39 (39.8)			
Yes	27 (17.0)	59 (60.2)			
Length of hospital stay (days)	8.00 (7.00, 10.00)	10.00 (9.00, 12.00)	−5.367	<**0**.**05**	−0.288

a,b No statistically significant differences were observed between left and right lung involvement, between left and bilateral involvement, or between right and bilateral involvement.

Bold values indicate statistically significant variables (*P* <0.05).

### 3.5 Univariate analysis of hematological indicators between the non-consolidation group and consolidation group in A2063G mutation-positive MPP children

Children in the pulmonary consolidation group had significantly lower ALB and UA levels than those in the non-consolidation group (all *P* < 0.05). Compared with children without pulmonary consolidation, those with consolidation presented an increased CLR (*P* < 0.05). No significant differences were observed between the children in the two groups for WBC, RBC, HB, PLT, NEU, LYM, MONO, EOS, CRP, PCT, IL6, LDH, CK, CKMB, ALT, AST, TP, GLB, BUN, Cr, NLR, MLR, PLR, PIV, or SII (all *P* > 0.05) (Table 4).

**Table 4:** Univariate analysis of hematological indicators between the non-consolidation group and consolidation group in A2063G mutation-positive MPP children (*n* = 257).

Variables	Non-consolidation group (*n* = 159)	Consolidation group (*n* = 98)	*Z*/*χ*^2^ value	*P* value	Effect size
WBC (×10^9^/L)	7.69 (5.99, 9.06)	6.90 (5.71, 8.46)	−1.854	0.064	−0.100
NEU (×10^9^/L)	4.64 (3.47, 5.66)	4.40 (3.35, 5.59)	−1.053	0.292	−0.057
LYM (×10^9^/L)	1.93 (1.39, 2.67)	1.88 (1.38, 2.33)	−1.148	0.251	−0.062
MONO (×10^9^/L)	0.56 (0.41, 0.77)	0.56 (0.40, 0.78)	−0.106	0.915	−0.006
EOS (×10^9^/L)	0.07 (0.03, 0.15)	0.05 (0.04, 0.15)	−0.643	0.520	−0.035
CRP (mg/L)	9.60 (5.20, 20.60)	14.20 (6.19, 25.90)	−1.929	0.054	−0.104
PCT (ng/mL)	0.10 (0.06, 0.16)	0.12 (0.07, 0.18)	−1.126	0.260	−0.060
IL6 (pg/mL)	16.78 (8.92, 24.49)	17.34 (11.90, 25.84)	−1.487	0.137	−0.080
NLR	2.38 (1.58, 3.33)	2.38 (1.70, 3.27)	−0.121	0.904	−0.006
MLR	0.28 (0.21, 0.37)	0.30 (0.22, 0.39)	−1.123	0.262	−0.060
PLR	143.80 (102.29, 186.14)	140.00 (103.78, 185.88)	−0.133	0.894	−0.007
CLR	5.18 (2.20, 13.13)	7.71 (3.30, 16.97)	−2.007	**<0** **.** **05**	−0.108
PIV	347.56 (196.29, 582.34)	310.97 (192.48, 572.64)	−0.835	0.404	−0.045
RBC (×10^12^/L)	4.57 (4.35, 4.91)	4.54 (4.35, 4.83)	−0.974	0.330	−0.052
HB (g/L)	124.00 (118.00, 130.00)	125.50 (120.00, 132.00)	−1.600	0.110	−0.086
PLT (×10^9^/L)	271.00 (223.00, 345.00)	261.00 (196.75, 310.25)	−1.880	0.060	−0.101
CK (U/L)	115.40 (79.50, 163.90)	105.00 (76.08, 171.80)	−0.334	0.738	−0.018
CKMB (U/L)	23.60 (19.14, 29.10)	22.10 (18.40, 28.50)	−1.648	0.099	−0.088
LDH (U/L)	313.10 (275.60, 353.60)	293.70 (262.45, 352.32)	−1.393	0.164	−0.075
ALT (U/L)	14.10 (11.40, 17.50)	13.20 (10.82, 16.73)	−1.425	0.154	−0.076
AST (U/L)	31.20 (25.60, 38.10)	30.40 (25.70, 34.02)	−1.016	0.310	−0.055
TP (g/L)	69.00 (67.10, 71.70)	68.50 (66.95, 70.42)	−0.969	0.332	−0.052
ALB (g/L)	41.44 ± 2.67	40.35 ± 2.75	3.171	<**0**.**05**	0.402
GLB (g/L)	27.97 ± 3.95	28.41 ± 3.37	−0.911	0.363	−0.139
BUN (mmol/L)	3.40 (2.80, 4.00)	3.20 (2.48, 3.60)	−1.959	0.050	−0.105
Cr (μmol/L)	36.85 ± 9.84	37.15 ± 8.54	−0.254	0.800	−0.033
UA (μmol/L)	274.00 (217.10, 324.00)	245.60 (198.68, 297.43)	−1.992	<**0**.**05**	−0.107

Bold values indicate statistically significant variables (*P* <0.05).

### 3.6 Multivariate logistic regression analysis of risk factors for pulmonary consolidation in A2063G mutation-positive MPP children

To further adjust for potential confounders and ensure model stability, variables with *P* < 0.10 in univariate analysis were entered into the multivariate logistic regression model. These variables included age, sex, fever duration, time from disease onset to treatment, coinfection, and relevant laboratory indicators (WBC, PLT, CRP, CKMB, ALB, BUN, and UA). In addition, peak temperature was excluded from the final multivariable model due to severe multicollinearity with fever duration (variance inflation factor >5), to ensure the stability and reliability of the regression model.

Tolerance values of all independent variables ranged from 0.647 to 0.911, and variance inflation factors (VIF) ranged from 1.097 to 1.546. According to conventional criteria (tolerance >0.1, VIF <2), no significant multicollinearity was observed among the independent variables (Table 5).

**Table 5:** Multivariate logistic regression analysis of risk factors for pulmonary consolidation in A2063G mutation-positive MPP children (*n* = 98).

Variables	*B*	SE	Wald *χ*^2^	*P* value	OR	95% CI
Age (month)	0.01	0.004	4.809	**<0** **.** **05**	1.010	1.001–1.019
sex	0.050	0.286	0.030	0.861	1.051	0.600–1.841
Duration of fever (days)	0.055	0.056	0.972	0.324	1.057	0.947–1.179
Time from onset to macrolide use (days)	−0.016	0.040	0.167	0.682	0.984	0.910–1.064
Co-infection	0.121	0.136	0.795	0.373	1.129	0.865–1.474
WBC (×109/L)	−0.006	0.058	0.011	0.917	0.994	0.888–1.113
PLT (×10^9^/L)	−0.001	0.002	0.516	0.472	0.999	0.995–1.002
CRP (mg/L)	0.003	0.007	0.173	0.678	1.003	0.989–1.018
CKMB (U/L)	−0.005	0.017	0.08	0.778	0.995	0.963–1.028
ALB (g/L)	−0.133	0.053	6.293	**<0** **.** **05**	0.875	0.789–0.971
BUN (mmol/L)	−0.192	0.156	1.5	0.221	0.826	0.608–1.122
UA (μmol/L)	−0.003	0.002	2.735	0.098	0.997	0.993–1.001

Note: Logistic regression results are presented as coefficient (B), standard error (SE), Wald *χ*^2^, *P* value, odds ratio (OR), and 95% confidence interval (95% CI).

Bold values indicate statistically significant variables (*P* <0.05).

**Figure 2:**
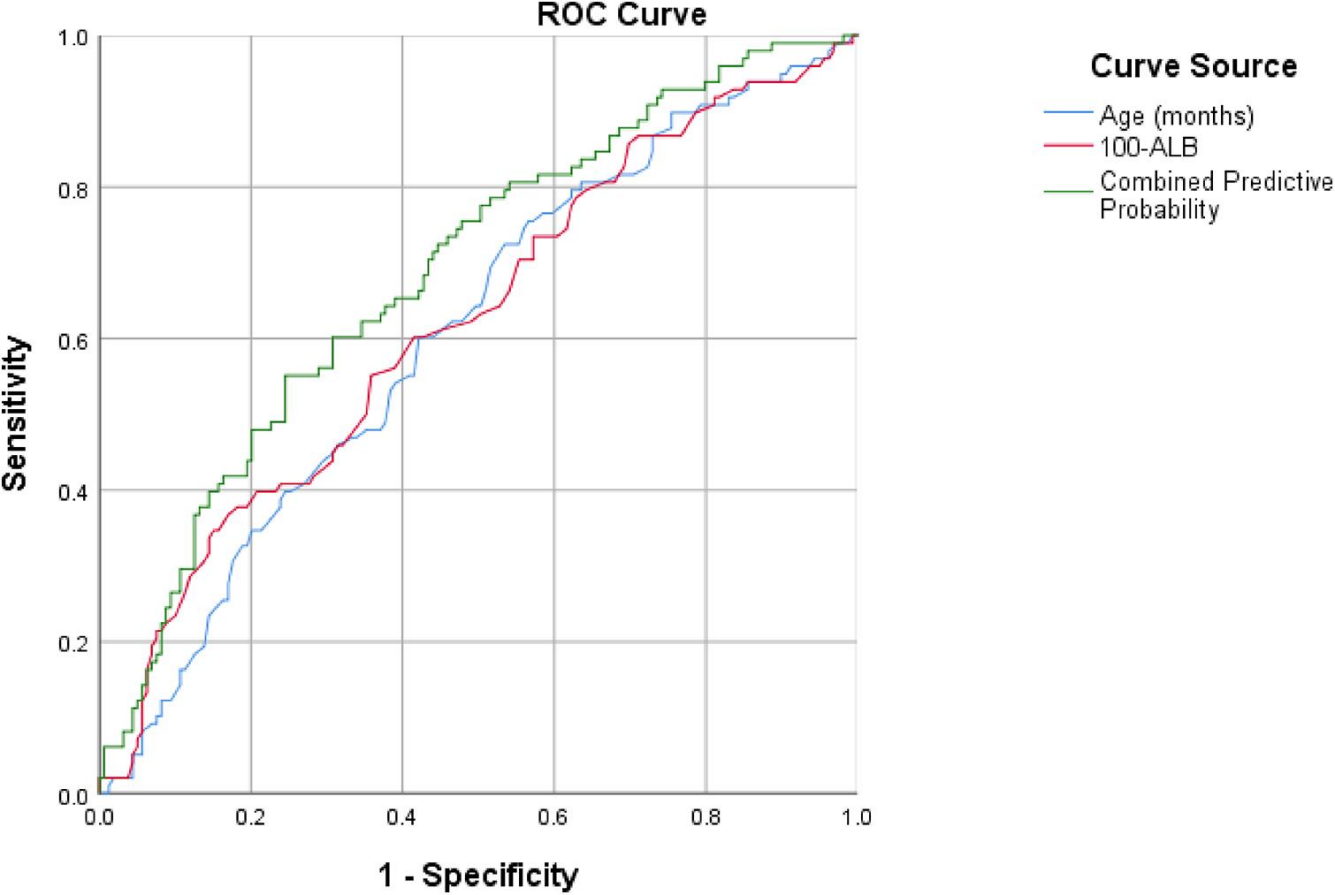
Receiver operating characteristic curves for predicting pulmonary consolidation A2063G mutation-postive MPP patients.

Multivariate logistic regression analysis identified age and ALB as independent factors associated with pulmonary consolidation (both *P* < 0.05). The Hosmer–Lemeshow test showed *χ*^2^ = 2.766, df = 8, *P* = 0.948, suggesting satisfactory model fit. The Nagelkerke pseudo-*R*^2^ was 0.133, indicating that the model accounted for 13.3% of the variance in pulmonary consolidation. Further assessment of predictive performance demonstrated that age, ALB, and the combined predictive model based on multivariate logistic regression all exhibited acceptable discriminatory ability, with the combined model outperforming individual indicators (all *P* < 0.05). The receiver operating characteristic (ROC) curves are displayed in Figure 2. The optimal cutoff values were 76.5 months for age and 39.15 g/L for ALB, respectively. The area under the curve (AUC) of age, 100-ALB, and their combined prediction model were 0.607, 0.620, and 0.686, with corresponding sensitivities of 0.725, 0.367, and 0.551, and specificities of 0.465, 0.830, and 0.755, respectively (Table 6).

**Table 6:** ROC curve cutoff values for risk factors associated with the development of pulmonary consolidation in children with A2063G mutation.

Variables	Cutoff Value	Sensitivity	Specificity	Youden index	AUC	*P*-value	95% CI
Age (month)	76.5	0.725	0.465	0.190	0.607	0.004	0.537–0.677
100-ALB	60.85	0.367	0.830	0.198	0.620	0.001	0.550–0.691
Combined predictive probability	0.44	0.551	0.755	0.306	0.686	<0.001	0.620–0.753

100-ALB is an inverse-transformed variable calculated as 100 − serum albumin (g/L). This transformation was applied in ROC curve analysis to align the variable direction with clinical risk interpretation, where lower albumin levels correspond to an increased risk of pulmonary consolidation. The original unit of albumin remains g/L throughout the study.

## 4 Discussion

In recent years, MPP in China have shown periodic epidemics with marked temporal and geographical variations. Previous studies have suggested that shifts in the proportion of distinct MP genotypes could be a key driver of its epidemic fluctuations (14, 17). In the present study, the detection rate of A2063G mutant strains was remarkably high, and these strains exhibited distinct clinical differences. This implies that molecular epidemiological shifts in MP may affect not only epidemic dynamics but also the clinical outcomes of children. Continuous genotyping and resistance monitoring are therefore essential for understanding MPP epidemiology and optimizing prevention and treatment strategies.

The incidence of pulmonary consolidation among A2063G mutation-positive cases was 38.1%. Compared with children having no pulmonary consolidation, children with pulmonary consolidation were older, had longer duration of fever, higher peak temperature, longer hospital stay, higher rates of severe pneumonia and CLR, but lower rates of bilateral lung inflammation, ALB, and UA. Multivariate logistic regression analysis showed that age and ALB were independent risk factors for pulmonary consolidation. On one hand, each 1-month increase in age was associated with an approximately 1% higher risk of pulmonary consolidation. This finding is consistent with previous studies (18, 19), indicating that older children are more prone to pulmonary consolidation, possibly because their more mature immune systems tend to trigger excessive inflammatory responses upon MP infection. On the other hand, ALB acted as a protective factor, with lower levels corresponding to an increased risk of pulmonary consolidation. Previous studies have confirmed that hypoalbuminemia is an independent risk factor for pulmonary consolidation (20), which may be attributed to excessive inflammatory response, increased ALB consumption, and elevated vascular permeability during severe infection. In this study, peripheral blood composite indices, which mainly reflect systemic inflammation, failed to effectively predict the occurrence of pulmonary consolidation. Therefore, in clinical practice, early risk assessment should combine relevant basic laboratory indicators with patient age, which is of great importance for preventing pulmonary consolidation.

Using throat swab tNGS, this study found a high resistance rate of 74.1%, with all resistant strains carrying the A2063G mutation. This aligns with both domestic and international reports (21–23), emphasizing the widespread and severe macrolide resistance in East Asia. Notably, the 257 A2063G mutation-positive children exhibited more pronounced abnormalities in cough prevalence, pulmonary consolidation rate, and several laboratory markers (higher GLB, lower LYM and ALB) than the mutation-negative children. However, the incidence of severe pneumonia was not significantly increased, which is consistent with some previous studies (24, 25). These findings suggest that while the A2063G mutation may exacerbate local pulmonary inflammation, it does not necessarily lead to overall clinical deterioration. In clinical practice, treatment strategies should be formulated based on local drug resistance surveillance data and individual patient characteristics, rather than directly extrapolating results from other regions. Early identification of A2063G mutation-positive strains and timely initiation of appropriate, targeted regimens can help avoid unnecessary or prolonged use of macrolides, which in turn reduces selection pressure for the emergence and spread of further drug resistance. Furthermore, mutation-positive children demonstrated consistently elevated peripheral blood composite indices (NLR, MLR, PLR, PIV, SII), reflecting a heightened systemic inflammatory response. In resource-limited settings lacking molecular testing, these indices may serve as surrogate markers for inferring resistance and supporting treatment decisions.

Taken together, divergent findings across studies indicate considerable heterogeneity in the clinical phenotypes of drug-resistant MPP. Accordingly, precision diagnosis and treatment should be guided by regional drug resistance profiles and individual patient characteristics to improve cure rates and reduce the risk of further antimicrobial resistance. Our results underscore the importance of integrating molecular testing, routine laboratory markers, and individual clinical features for personalized risk assessment and therapeutic decision-making, which may help optimize the management of children with drug-resistant MPP.

## 5 Limitations

This study was a single-center retrospective analysis with a limited sample size, so the generalizability of its conclusions needs to be validated in a larger population. Although tNGS was used for etiological detection, throat swab specimens may have been influenced by colonizing bacteria to some extent, which remains a limitation. In addition, this study mainly explored the relationship between the A2063G mutation and pulmonary consolidation based on clinical characteristics, and there is still a lack of experimental verification of the underlying mechanisms. Future multi-center, large-sample, prospective studies should be conducted, combined with basic experiments to investigate its molecular mechanisms, thereby further enhancing the clinical applicability of the findings.

## 6 Conclusions

In summary, the study retrospectively reviewed the clinical characteristics, imaging findings, and laboratory parameters of 347 children with MPP, and investigated risk factors for pulmonary consolidation in drug-resistant MPP. The results showed that age and ALB levels were independent risk factors for pulmonary consolidation, and the clinical phenotypes of drug-resistant MPP exhibited significant heterogeneity. Clinically, it is necessary to formulate personalized diagnosis and treatment strategies based on regional drug resistance patterns and individual patient conditions, and integrate molecular detection and conventional laboratory indicators to optimize the management of children with drug-resistant MPP. This study provides a theoretical basis and practical reference for improving the clinical diagnosis and treatment level of drug-resistant MPP and reducing the risk of drug resistance. These results are preliminary and necessitate prospective validation prior to therapeutic application.

## Data Availability

The datasets presented in this article are not readily available because the dataset generated and analyzed during the current study is subject to the following restrictions: 1. Patient privacy protection restriction: The dataset contains sensitive personal and clinical information of pediatric patients (e.g., demographic data, laboratory test results, imaging findings). In accordance with the ethical approval requirements of the Ethics Committee of Guangzhou Red Cross Hospital and relevant national regulations on medical data privacy, the raw dataset cannot be publicly shared or distributed to third parties to avoid compromising patient confidentiality. 2. Research purpose restriction: Any use of the de-identified dataset (if applicable) is restricted to academic research purposes only. Commercial use, non-research-related applications, or any use that violates medical ethics and data protection laws are strictly prohibited. 3. Authorized access restriction: Qualified researchers may request access to the de-identified dataset for legitimate research purposes after submitting a formal application to the research team and obtaining approval from the Ethics Committee of Guangzhou Red Cross Hospital. Access will be granted only upon signing a data use agreement to ensure compliance with all relevant restrictions and safeguards. Requests to access the datasets should be directed to not applicable.
